# The impact of paternal age on cumulative assisted reproductive technology outcomes

**DOI:** 10.3389/fmed.2023.1294242

**Published:** 2024-01-08

**Authors:** Clemence Farabet, Paul Pirtea, Achraf Benammar, Dominique De Ziegler, Claire Marchiori, Alexandre Vallée, Jean-Marc Ayoubi

**Affiliations:** Department of Obstetrics, Gynecology and Assisted Reproduction, Hospital FOCH, Suresnes, France

**Keywords:** retrospective cohort study paternal age, ART, fresh embryo transfer, frozen embryo transfer, age

## Abstract

**Objective:**

To investigate the impact of paternal age on cumulative live birth rate in ART.

**Design:**

Retrospective single-center cohort study.

**Patient(s):**

All female patients aged 18–43 years and male patients aged 18–60 years, who performed their first ART cycle between January 2018 and December 2020, were included.

**Main outcome measure(s):**

The primary outcome, cumulative live birth rate (cLBR), was estimated following fresh or frozen embryo transfers issued from an ART cycle. Secondary outcomes included the cumulative pregnancy rate (cPR) and miscarriage rate. Subgroup analyzes were performed as follows: men <45 and ≥ 45; female <35, 35–38, and > 38 years.

**Result(s):**

A total of 2,358 couples were included in this study. The sperm quantity of male patients within both age groups was divided in two groups: normal and abnormal, which were found to be in significantly equal proportions. There were significantly fewer current smokers in the male group ≥45. The cPR was 0.5301 in the group <45 and 0.3111 in the group ≥45, with a *p-value* <0.001. Analysis according to the female age revealed that, in the female group >38, the cLBR rate was 0.26 for men <45 and 0.19 for men ≥45, with a *p-value* of 0.061. Additionally, the cPR was 0.34 in the male group <45 and 0.21 in the group ≥45, with a *p-value* <0.001. In the female group between 35 and 38 years of age, the cLBR was 0.44 in the male group <45 and 0.3 in the male group ≥45, with a *p-value* of 0.031. The cPR was 0.49 in the male group <45 and 0.34 in the group ≥45, *p* = 0.036. Within the female group <35, we observed non-significant results. The miscarriage rate results were not significantly different for women ≤38.

**Conclusion:**

According to the results from our study, male age ≥ 45 has a significant impact on cumulative ART outcomes.

## Introduction

In assisted reproductive technologies (ART), women’s age has long been recognized as the most predictive factor. This effect parallels the fact that maternal age is strongly associated with couples’ chances of natural conception. In ART, increasing maternal age—notably, beyond 35 years—is associated with increased risk of embryo aneuploidy, implantation failure, and miscarriage (1). Conversely, the age of the male partner has received lesser interest. Demonstrating a relationship between paternal age and poor ART outcomes is a challenging task due to the inherent difficulty at singling out the father’s age from the maternal one or other confounders. Some studies reported a positive relationship between increased paternal age and poor ART outcomes (2–5); however, no clearly defined age threshold was validated in these studies. Some authors reported lower pregnancy rates (PRs) and livebirth rates (LBRs) after controlling for maternal age and other confounders for men over 46 years (6) or over 50 years (7) of age, compared to younger men. However, when restricted to women aged <35 years, there was no significant difference between the two male groups (6).

Mc Pherson et al. (8) reported a decrease in LBR among couples whose female partner was >35 years and male partner was >40 years of age. They hypothesized that the negative effect stemmed from an increase in sperm DNA fragmentation and alteration of oocyte’s cytoplasm DNA repair mechanisms in these couples.

Poorer spermatic parameters as well as changes in the genetic and epigenetic statuses of spermatozoa in aging men have been found as a possible causative factor in the overall decline of ART outcomes (3, 9–12). The known effects of paternal age on reproduction include an alteration of sperm parameters and genomic alteration characterized by spontaneous mutations, sperm DNA fragmentation, and telomere lengths (13).

In contrast, other studies have found no significant impact of paternal age on ART results (9, 14, 15). In a study involving 278 couples, Nijs et al. observed no difference in fertilization rates, pregnancy rates, and LBRs related to paternal age when controlling for maternal age (16). However, these authors excluded men with severe oligo-astheno-teratozoospermia (OAT), those who used testicular sperm for ICSI, and cases involving preimplantation genetic testing while this represents a significant portion of the patients managed in IVF (16).

As women seeking ART are progressively older, the role of male age gains more interest. Nowadays, the average age of conception for a couple is continuously increasing year after year. Regarding paternal age, according to a 2017 review, fathers at the time of their first child’s birth are on average 3.5 years older than 40 years ago (17). Over the same period, the percentage of fathers over 49 years old has doubled (17).

This discrepancy between reports of poorer ART outcomes when the male partner is older and the observation of constant – age independent – donor egg ART results has led us to question whether male age might have a more important effect in older women. In donor egg ART, oocyte donors are generally young, and this may negate any impact of male’s age.

Indeed, success rates of donor egg ART remain constant until the age of 50 years even though, in general, the male partner is slightly older than his spouse (2–4). This has greatly contributed to the belief that the role of the age of the male partner is of menial importance, if any, in ART.

To test this hypothesis, we opted to study the impact of male age on ART outcomes as a function of the female’s age. We therefore decided to analyze the possible impact of paternal age on cumulative ART outcomes in different age categories of men and women.

## Materials and methods

We conducted a retrospective cohort study including all IVF or ICSI cycles performed at Foch Hospital from January 2016 to December 2020. We included all women of ages ranging from 18 to 43 years and men from 18 years to 59 years, who underwent their first ART cycle at Foch hospital. We excluded those couples where sperm was obtained from a testicular biopsy or a donor and those with a number of oocytes retrieved strictly inferior to 4. We did not include PGT cases. We characterized men by sperm parameters and the use of tobacco. Spermatic alterations are defined as follows: low OAT (concentration < 10 millions/ml), severe OAT (concentration < 5 millions/ml), extremely severe OAT (concentration < 1 million/ml). We collected AMH levels and total dose of gonadotropin used in controlled ovarian stimulation (COS). Biochemical pregnancies were excluded. At each embryo transfer, the embryo with the best morphology as determined by Gardner scale was transferred first. Cumulative results for the pregnancy rate (PR) and live birth rate (LBR) were calculated after all embryos were transferred or until the obtention of the first live birth. The main outcome was the cumulative live birth rate (cLBR) and the secondary outcomes were the cumulative pregnancy rate (cPR) defined by the presence of cardiac activity at the time of the first ultrasound approximatively 7–8 weeks after the embryo transfer. Additionally, the miscarriage rate was assessed, defined as any pregnancy in which cardiac activity stopped before 12 weeks of gestation.

For the female group, we opted to use the SART group ages, whereas for the male group, after reviewing the literature, we retained the cut-off age of 45.

## Ethical approval

Given the retrospective nature of this study, the access and processing of patient data was approved by the CERF – French Research Ethic Committee IRB 00012437 from 13/04/2022. All methods were performed in accordance with the relevant guidelines and regulations.

## Patient treatment

The ART treatment followed FOCH AMP routine protocols. COS was performed using highly purified urinary menotropins (hMG) and recombinant FSH (rFSH). For OS, individually set doses of hMG and/or rFSH were used, ranging from 150 to 600 IU/day in an GnRH-antagonist protocol. The development of ovarian follicles was monitored by transvaginal ultrasonography beginning on the seventh day of OS. If required, hormonal doses were adjusted to generate an optimal ovarian response. The gonadotropin-releasing hormone (GnRH) antagonist (Cetrorelix^®^ 0.25 mg Merck France) was introduced systematically on the sixth day of OS.

Final oocyte maturation was triggered using a combination of human chorionic gonadotropin (hCG) (Ovitrelle^®^ 250 μg Merck France) with 2 ampules of GnRH agonist, triptoreline Decaptyl^®^ (0.1 mg Ipsen France), or triptoreline only GnRH 0.3 mg, if there was a risk of an ovarian hyperstimulation syndrome, when ≥3 mature follicles of ≥18 mm were confirmed by vaginal ultrasound. Transvaginal oocyte retrieval (TVOR) was performed 36 h after ovulation trigger. The thickness of the endometrium had to be greater than 7 mm, with progesterone less than 1.5 ng/mL on the day of the ovulation trigger, to perform a fresh embryo transfer. If there was a risk of hyperstimulation, or an incidental discovery of polyps, the entire cohort of embryos was frozen. Mature oocytes were fertilized using ICSI, if considered necessary. Blastocyst embryos were graded accordingly. Embryos were cultured in 90% N_2_, 5% O_2_, and 5% CO_2_ mixture at 37°C. Blastocysts were analyzed on Day 5 and/or 6 and graded. Excellent and good-quality embryos were defined as B3, B4, or B5 embryos ≥4 BB (AA, AB, BA, BB). Embryos were vitrified using High Security straws (Cryo-Bio-System) combined with DMSO-EG-S as cryoprotectants (Irvine Scientific Freeze Kit^©^ United states). The same kit was used for the warming process.

In the case of fresh embryo transfer, patients began vaginal progesterone Progestan^®^ (200 mg Bezins France, 1 capsule, 3 times a day) from the evening of the retrieval and oral estradiol Provames^®^ (2 mg Merus Luxembourg, 2 tabs BID) starting on the day of embryo transfer. The single or double embryo transfer was performed at Day 2 or 5, under transabdominal ultrasound guidance using a soft catheter.

In the case of a frozen embryo transfer, patients were seen approximately 1 month later. They received oral estradiol Provames^®^ provenance (2 mg, 1 tab BID from Day 1 to 4, then 1 tab in the morning and 2 in the evening from Day 5 to 9 then 2 tabs BID) thereafter.

Endometrial thickness was monitored on transvaginal ultrasound and serum progesterone was measured to rule out premature ovulation before initiation of progesterone treatment. Endometrial thickness had to be >7 mm, and progesterone less than 1.5 ng/mL. Progesterone administration consisted of a combination of subcutaneous injections of progesterone (Progiron^®^ Genevrier France) 25 mg once daily and 2 vaginal progesterone capsules BID (Progestan^®^) (200 mg) starting 5 days before the transfer. On the sixth day of progesterone administration, one or two warmed blastocysts were transferred. When more than one embryo was available, the choice was made based on morphological grading. The frozen embryo transfer was performed under transabdominal ultrasound guidance using a soft catheter. Daily estrogen and progesterone administration was continued until the pregnancy test. Hormone administration was continued until the expected lutheo-placental shift, at 9 weeks of gestation.

## Statistical analysis

Continuous data were described with median and 25th and 75th percentile due to their non-normal distribution, verified by the Shapiro–Wilk test (for age in men and women).

The cLBR, CPR, and miscarriage rate were expressed by quotations “0” and “1.” Furthermore, the mean and standard deviation were presented and tested by the Mann–Whitney test. The *p*-value < 0.05 was considered significant. All analyzes were performed using SAS software (version 9.4; SAS Institute, Carry, NC). We categorized paternal age into two groups: <45 years and > 45 years. To evaluate the effect modification by maternal age, we stratified maternal age < 35 years, 35–38, and > 38 years at the beginning of the cycle.

## Results

Our study included 2,358 couples who met the inclusion criteria. The median male age was 37.4 years, and the median female age was 35.3 years. The vast majority (86.6%) of reported cycles occurred with men aged <45 years, with only 13.4% of cycles involving men aged ≥45 years (Table 1). Sperm parameters were comparable between the two groups, even if abnormal semen parameters were more prevalent in the group ≥45 years; the difference was not statistically significant. Tobacco consumption at the time of the stimulation was more common in the group <45 years, and men in the group ≥45 years were more likely to be non-smokers or former smokers (Table 2).

**Table 1:** Repartition of male and female patients by age groups.

Male		<45	%	≥45	%
Female	<35	1,062	51.9	58	18.4
	35–38	494	24.3	61	19.3
	> 38	487	23.8	196	62.3

**Table 2:** Selected variable parameters: tobacco consumption (yes, no, or former smoker), sperm parameters (normal, moderate OAT, severe OAT, and extremely severe OAT), female age, female AMH, and gonadotrophin dose during the stimulation in the two male groups, <45 and ≥ 45 years of age.

Male age	<45		≥45		
	*N*/median	% or interquartile	*N*/median	% or interquartile	*p* value
Female age (MEAN)	35	[32–38]	39	[36–41]	<0.001
Male age (mean)	37	[33–40]	48	[46–51]	0.979
Tobacco consumption					0.039
No	1,161	61.27%	189	65.17%	
Yes	420	22.16%	46	15.86%	
Former smoker	314	16.57%	55	18.97%	
Sperm parameters					0.704
Normal	1,642	80.37%	252	80%	
Light Oat	169	8.27%	22	6.98%	
Severe OAT	133	6.51%	22	6.98%	
Extremely severe OAT	99	4.85%	19	6.03%	
Female parameters					
AMH	3.117	2.965 sd	2.513	2.394 sd	<0.001
Gonadotrophin dose	4,268	1937 sd	4,912	1983 sd	<0.001

Older paternal age was associated with older maternal age and therefore lower AMH and the use of higher doses of gonadotrophin (Table 2).

Table 3 shows the results of the analysis for the primary outcome according to paternal age. The cLBR for men ≥45 years of age (0.2857 ± 0.4525) was lower than that in the group <45 (0.4714 ± 0.4993), with a *p*-value of <0.001 and the results of the analysis for the secondary outcome cPR are also presented in the table. The cPR for men ≥45 years of age (0.311 ± 0.4637) was lower than that in the group <45 years of age (0.5301 ± 0.4992), with a *p*-value of <0.001.

**Table 3:** Results of the primary and secondary outcomes, which includes cLBR, cPR, and miscarriage rate in the two male groups, <45 and ≥ 45 years of age.

Male age	<45		≥45		*p* value
	*N* = 2043		*N* = 315		
	Mean	Std Dev	Mean	Std Dev	
cLBR	0.4714	0.4993	0.2857	0.4525	< 0.001
cpr	0.5301	0.4992	0.3111	0.4637	< 0.001
Miscarriage RATE	0.0788	0.269	0.041	0.199	0.02

Table 4 shows the results of the analysis of the live birth rate and pregnancy rate after the fresh embryo transfer, according to paternal age. The LBR for men ≥45 years of age (0.175 ± 0.395) was significantly lower than the LBR for men <45 years of age (0.331 ± 0.554), with a *p*-value of <0.001.

**Table 4:** Results (LBR and PR) after fresh embryo transfer.

Male age	< 45		≥ 45		*p* value
	Mean	Std Dev	Mean	Std Dev	
LBR	0.331	0.554	0.175	0.395	< 0.001
PR	0.376	0.58	0.213	0.461	< 0.001

Subsequently, the PR for men ≥45 years of age was significantly lower than the PR for men <45 years of age.

Table 5 shows the results of the analysis of the cumulative live birth rate and cumulative pregnancy rate after the frozen embryo transfer, according to paternal age. The cLBR for men aged ≥45 years (0.298 ± 0.485) was significantly lower than the cLBR for men aged <45 years (0.486 ± 0.531), with a *p*-value of <0.001.

**Table 5:** Cumulative results (cLBR and cCPR) after frozen embryo transfer only.

Male age	< 45		≥ 45		*p* value
	*N*	1,389	*N*	194	
	Mean	Std Dev	Mean	Std Dev	
cLBR	0.412	0.514	0.269	0.471	< 0.001
cPR	0.486	0.531	0.298	0.485	< 0.001

The cPR for men ≥45 years of age was significantly lower than the cPR for men <45 years of age.

Table 6 shows the results of the analysis of the live birth rate and clinical pregnancy rate after the first single frozen embryo transfer, according to paternal age. The LBR for men ≥45 years of age (0.189 ± 0.393) was lower than the LBR for men <45 years of age (0.292 ± 0.457), with a *p*-value of 0.015. The PR for men ≥45 years of age was significantly lower than the PR for men <45 years of age.

**Table 6:** Results (LBR and PR) after the first single frozen embryo transfer.

Type of ET	SET				
Male age	< 45		≥ 45		*p* value
	*N*	991	*N*	132	
	Mean	Std Dev	Mean	Std Dev	
LBR	0.292	0.457	0.189	0.393	0.015
PR	0.343	0.479	0.197	0.399	0.001

Table 7 shows the results of the analysis of the live birth rate and clinical pregnancy rate after the first double frozen embryo transfer, according to paternal age. The LBR for men ≥45 years of age (0.338 ± 0.625) was lower than the LBR for men <45 years (0.51 ± 0.698), with a *p*-value of 0.068. The PR for men ≥45 years was significantly lower than the PR for men <45 years of age.

**Table 7:** Results (LBR and PR) after the first double frozen embryo transfer.

Type of ET	DET				
Male age	< 45		≥ 45		*p* value
	*N*	398	*N*	62	
	Mean	Std Dev	Mean	Std Dev	
LBR	0.51	0.698	0.338	0.625	0.068
PR	0.61	0.735	0.403	0.639	0.039

After stratification on maternal age, as presented in Table 8, the cLBR was lower in the ≥45 age group (0.19 ± 0.39) than in the <45 age group (0.26 ± 0.44), with a *p*-value of 0.061 for women >38 years and for women between 35 and 38 years of age (0.3 ± 0.46 vs. 0.44 ± 0.5), with a *p*-value of 0.031. The cPR was also lower in the ≥45 age group (0.21 ± 0.41) than in the <45 age group (0.34 ± 0.47), with a *p*-value <0.001 for women >38 years and for women between 35 and 38 years (0.34 ± 0.48 vs. 0.49 ± 0.5), with a *p*-value of 0.036. When the analysis was restricted to cycles with maternal age < 35 years, the result was not statistically significant; the cLBR was 0.59 ± 0.49 in the group ≥45 years of age and 0.58 ± 0.49 in the group <45 years of age, with a *p*-value of 0.948; and the cPR was 0.62 ± 0.49 in the group ≥45 years of age and 0.64 ± 0.48 in the group <45 years of age, with a *p*-value of 0.785.

**Table 8:** Results of stratified analysis: cLBR, cPR, and miscarriage rate in the two male groups, < 45 and ≥ 45 years of age, stratified by female’s age < 35, 35–38, and > 38 years of age.

Male age		< 45	≥ 45	*p* value
cLBR	<35	0.58 (0.49)	0.59(0.49)	0.948
	35–38	0.44 (0.50)	0.30 (0.46)	0.031
	> 38	0.26 (0.44)	0.19 (0.39)	0.061
cPR	< 35	0.64 (0.48)	0.62 (0.49)	0.785
	35–38	0.49 (0.50)	0.34 (0.48)	0.036
	> 38	0.34 (0.47)	0.21 (0.41)	<0.001
Miscarriage rate	< 35	0.0847 (0.2786)	0.1034 (0.3072)	0.621
	35–38	0.0567 (0.2315)	0.0656 (0.2496)	0.779
	> 38	0.0883 (0.284)	0.0153 (0.1231)	<0.001

Regarding the secondary outcome, the miscarriage rate was found to be lower in the group aged ≥45 years (0.041 ± 0.199) compared to the group aged <45 years (0.0788 ± 0.269), with a *p*-value of 0.02 (Table 3).

For women >38 years of age, the miscarriage rate was 0.0153 ± 0.1231 in the group ≥45 years of age, and 0.0883 ± 0.284 in the group <45 years of age, *p* < 0.001. When the analysis was restricted to cycles 35–38, the miscarriage rate was higher in the group ≥45 years of age (0.0656 ± 0.2496) compared to the group <45 years of age (0.0567 ± 0.2315), with a *p*-value of 0.779; and same statement for women <35 years (0.1034 ± 0.3072 in the group ≥45 years vs. 0.0847 ± 0.2786 in the group <45, *p* = 0.621) (Table 8).

## Discussion

Our result indicates that, in fresh or frozen IVF or ICSI cycles performed at Foch hospital between 2016 and 2020, advanced paternal age (≥45 years old) was associated with lower cPR and cLBR. When the analysis was limited to women <35 years of age, results were not significantly influenced by paternal age. These findings suggest that oocytes from women <35 years of age could correct sperm anomalies affecting fertility in the case of advanced paternal age. Miscarriage rates were lower with advanced paternal age. When the analysis was restricted to women ≤38 years of age, a trend toward a reduction in the number of miscarriages appeared in the group <45 years but not in a statically significant way.

Our findings are consistent with the results of several studies. In a retrospective cohort study, among 77,209 fresh IVF cycles, compared with paternal age < 45 years, paternal age ≥ 46 years was associated with a lower likelihood of pregnancy per ART cycle (adjusted risk ratio [aRR] 0.81; 95% confidence interval [CI] 0.76–0.87) and per transfer (aRR 0.85; 95% CI 0.81–0.90), as well as a lower likelihood of live birth per cycle (aRR 0.76; 95% CI 0.72–0.84) and per embryo transfer (aRR 0.82; 95% CI 0.77–0.88) after controlling for maternal age and other potential confounders. When restricted to women aged <35 years, no significant differences in the rates of live birth or miscarriage among couples in which the men were aged ≥45 years emerged compared with those aged ≥46 years (6).

A retrospective cohort study conducted in 2019 analyzed 2,425 cycles of couples. There was a gradual negative effect of male age and female age on live birth as odds ratios (OR) with 95% CI for each additional year of age (OR-male age: 0.96 [0.94–0.98]; OR-female age: 0.90 [0.88–0.93] *p* < *0.001*). Secondary outcomes showed a significant reduction in the odds of clinical pregnancy (OR-male age: 0.97 [0.96–0.99]; OR-female age: 0.92 [0.89–0.94] *p* < *0.001*) and an increase in the odds of miscarriage with greater age: male age (OR: 1.05 [1.01–1.08]; *p* = *0.002*) and female age (OR: 1.11 [1.05–1.18]; *p* < *0.001*) (7). Regarding the increase of miscarriages after 40 years of male age, advanced paternal age was found to be associated with an increased risk of miscarriage, independent of chosen factors (18, 19).

Advanced paternal age has also been associated with higher miscarriage rates among pregnancies using ART procedures. However, other studies have not consistently demonstrated an association between older paternal age, ART procedures, and miscarriages. These different studies are in majority retrospective, the population of older men was scarce, and they potentially impacted the pregnancy outcomes by excluding women with advanced maternal age (20).

Even though the direct consequence of advanced paternal age on miscarriage is less obvious, it is biologically possible that a higher number of genetic and epigenetic sperm abnormalities in older men could have an influence on miscarriages (19).

More recently, several reports have investigated the parental origins of chromosomal imbalances. Bonus et al. evaluate the relationship between paternal factors and embryonic aneuploidy of paternal origins, specifically paternal age. There was no statistically significant correlation between paternal age and incidence of aneuploidy of paternal origins. However, it is interesting to see a trend in the association of aneuploidy of paternal origin with increasing paternal age. Particularly in the case of age, the *p*-value closely approached significance (21).

Several confounding factors must be taken into account when evaluating the influence of paternal age on ART results beyond the fact that this is a study using genetically unscreened embryos. First, male infertility is an significant factor to consider, independently of male age, as it has been shown that the severity of male factor influences ART outcomes (22). Second, environmental considerations, such as the use of alcohol, smoking, medications with a gonadotoxic effect, obesity, and other comorbidities that may impact ART outcomes (23) must be considered. Third, maternal age as a confounding factor should be considered, as the age of women has a prominent role on ART outcomes. The perfect model to independently assess paternal age impact would be to use oocytes from oocyte donors, in order to avoid a bias related to oocyte quality. Indeed, the oocyte donor population are young women without fertility problems. Unfortunately, few studies evaluated the impact of paternal age in the oocyte donor population.

Begueria et al. in an egg donation model with ICSI as fertilization method found that male age was not associated with any pregnancy outcome: biochemical pregnancy rate (RR: 1.0; 95% ci: 0.96–1.05), miscarriage rate (RR: 1.06; 95% ci: 0.94–1.03), ongoing pregnancy rate (RR: 0.98; 95% ci: 0.94–1.033), and LBr (RR: 0.98; 95% ci: 0.94–1.03) (9).

In their study, Dviri et al. (20) evaluated over 3,000 embryos derived from cycles using oocyte donors from women aged <33 years and stratified by paternal age (<39, 40–49, >50). No association was found between paternal age and aneuploidy rates. Advanced paternal age > 50 compared with younger paternal age was associated with a lower fertilization rate and an increased rate of segmental aberration (24). This is somewhat in concordance with recent retrospective studies, reporting that advanced paternal age was associated with a reduced embryo quality, a reduction of fertilization and pregnancy rates, and hence a reduction in pregnancy and live birth rates (20).

The clinical relevance of increased segmental aneuploidy in older men has yet to be explored. Sperm DNA fragmentation is more common in older men (25), and it could potentially be an explanation for why segmental changes affect the paternal chromosomes at older ages. Further studies evaluating DNA fragmentation and paternal segmental aneuploidy would be worthwhile (26).

Our study also holds a result that we cannot readily interpret. Miscarriage rates were lower with advanced paternal age. When the analysis was restricted to women <38 years of age, a trend toward a reduction in the number of miscarriages appeared in the age group <45 years, but not in a statically significant way. These findings, unexpectedly, may only represent a statistical fluke; however, for sake of thoroughness, they are reported here.

This study bears limitations: the miscarriage rate found in the group of men <45 years is higher than in the group of men over 45 years of age. By analyzing the stratified study on female’s age, we can notice that this effect is only present in women >38 years of age. This difference, while counterintuitive, could be explained through the repartition of ages within each category. Of the 487 women over the age of 38 years were associated with young men (23.8%), while 196 women were associated with older men (62.3%). Additionally, by observing the standard deviation, we notice a higher difference between the two groups of men (≥45 years and < 45 years) in the category of women >38 years than in the other group of women. The data have been continuously verified in the male population without any split between the two groups and with a cut-off at 45 years of age; however, this difference is not highlighted in the observed miscarriage rate. Furthermore, another data-check has been performed using a different cut-off at 40 years instead of 45 years, and once again, the difference in the miscarriage rate has not been highlighted.

Another notable limitation of our study was the uneven distribution of cases analyzed in the paternal age category >45 years: few cases were associated with women <35 years compared to the number of men >45 years associated with women >38 years. However, these data were representative of our population.

Another limitation was the lack of PGT-a use, which would have allowed us to filter the chromosomic abnormalities and would have rendered our analysis more precise.

There was a lack of data on women’s BMI in our database, so we did not include it in the analysis.

One notable strength of our study was the large statistical analysis performed on 2,358 couples and the fact that we were able to stratify the analysis based on age within the ART groups.

## Conclusion

In couples undergoing ART procedures for infertility, advanced paternal age is associated with a lower cPR and cLBR in ART. We assert that our data add relevant information for understanding certain ART failures, emphasizing the role of considering both female and male ages when assessing ART outcomes. The psychological and ethical impacts of advanced paternal age should also be discussed, as is the case with egg donation for women of advanced age.

### Impact

We report that male age ≥45 has a significant impact on cumulative ART outcomes, an effect particularly pronounced in women over 38 years of age.

## Data availability statement

The raw data supporting the conclusions of this article will be made available by the authors, without undue reservation.

## Ethics statement

The access and processing of patient data was approved by the CERF – French Research Ethic Committee IRB 00012437 from 13/04/2022. Written informed consent for participation was obtained from all subjects that were included in our study. The studies were conducted in accordance with the local legislation and institutional requirements.

## Author contributions

CF: Writing – original draft, Conceptualization, Methodology. PP: Conceptualization, Methodology, Supervision, Writing – review & editing. AB: Data curation, Formal analysis, Investigation, Writing – review & editing. DZ: Conceptualization, Methodology, Writing – review & editing. CM: Data curation, Formal analysis, Writing – review & editing. AV: Formal analysis, Writing – review & editing. J-MA: Methodology, Project administration, Resources, Supervision, Validation, Writing – review & editing.
